# Computed tomography long‐term follow‐up of a facial fracture involving the orbit and paranasal sinus

**DOI:** 10.1002/evj.70224

**Published:** 2026-06-24

**Authors:** A. Kalinovskiy, C. Lischer, J. C. Eule, K. Thieme, K. Mählmann

**Affiliations:** ^1^ Equine Clinic Freie Universität Berlin Berlin Germany; ^2^ Unit for Ophthalmology Veterinary Hospital, Freie Universität Berlin Berlin Germany

**Keywords:** bone resorption, horse, mucocele, orbital rim fracture, sinus

## Abstract

**Background:**

Fractures of the facial bones involving the orbit are challenging conditions that require surgical treatment.

**Objectives:**

To describe preoperative and postoperative clinical and computed tomographic findings of an orbital rim fracture repaired using a reconstruction plate.

**Study Design:**

Case report.

**Methods:**

A 14‐year‐old Haflinger mare was presented with an open, depressed and comminuted fracture involving the frontal, nasal, lacrimal and maxillary bone, sinus system and the medial aspect of the orbital rim and wall on the right side, as diagnosed by CT. Fracture repair included orbital rim reconstruction with a pre‐shaped 12‐hole 3.5‐mm LCP‐reconstruction‐plate and repositioning/fixation of several fragments, including one large frontal nasal bone fragment, with cerclage wire sutures. Despite an implant infection postoperatively, the fracture healing assessed by CT 10 weeks later was considered good, and the reconstruction plate was removed at the same time.

**Results:**

CT follow‐up 1 year after fracture fixation revealed excellent restoration and healing of the orbital rim and wall. Complete resorption of the large repositioned frontal/nasal bone fragment was observed, resulting in a skin‐covered bone defect adjacent to the right sinus system. CT imaging also suggested a potential mucocele formation within the right sinus system. Both findings were clinically inapparent and identified solely on CT examination.

**Conclusions:**

An excellent long‐term outcome was achieved in this orbital fracture repaired with application of a pre‐shaped 3.5‐mm LCP‐reconstruction plate, despite sinus communication, severe comminution and later implant infection, as assessed by CT. In conclusion, CT proved highly valuable for a systematic approach to fracture treatment and perioperative assessment of facial fractures in the present case. Several findings that had not been previously described could be characterised for the first time based on CT assessment. However, the long‐term clinical relevance of the described findings needs further evaluation.

## 1 INTRODUCTION

Fractures involving the orbital region pose significant challenges due to the potential involvement of the globe, adnexa, neurovascular structures, nasolacrimal duct, and sinuses.^1^, ^2^, ^3^, ^4^, ^5^

Computed tomography would be the gold standard for diagnosis in all facial/orbital fractures.^6^, ^7^ Surgical treatment is indicated in cases of globe impingement to restore functional anatomy and improve cosmetic outcome. Depending on the fracture configuration, repair techniques may include simple repositioning, stabilisation with cerclage wire or polydioxanone sutures, as well as plate fixation.^2^, ^3^, ^8^, ^9^ The prognosis with fractures involving the orbit or sinus systems is generally good with appropriate surgical management.^5^, ^7^, ^10^

Potential complications associated with sinofacial fractures include chronic sinusitis, the development of a sinocutaneous fistula or sequestrum, and facial deformity. In cases where fracture repair involves the use of implants, surgical site infection (SSI) represents an additional risk.^3^, ^7^ Furthermore, when the orbit is affected, complications may encompass uveitis, corneal ulceration, globe rupture, hyphema, injury to intraocular structures, blindness, nasolacrimal duct occlusion, and the development of enophthalmos/exophthalmos.^1^, ^7^

This report provides a detailed description of the preoperative and postoperative clinical and CT‐findings of an open, depressed and severely comminuted facial fracture involving the sinus system and orbit in a horse. The fracture was repaired using a reconstruction plate and cerclage wire, and follow‐up CT scans were performed over a period of up to 1 year. Although orbital fractures with sinus involvement and their management have already been described,^1^, ^2^, ^3^, ^4^, ^11^ this report is the first detailed description of this specific surgical procedure. Additionally, long‐term CT findings after fracture repair in this region were not previously reported in horses.

## 2 CLINICAL REPORT

### 2.1 History, clinical and sonographic findings

A 14‐year‐old Haflinger mare was referred with a right‐sided depressed skull fracture following a kick injury 1 day earlier. On admission, the mare was bright and responsive, and physical, haematological, and biochemical findings were unremarkable. Marked depression of the medial orbital rim and adjacent frontal, nasal, and maxillary bones was present with oedema, subcutaneous emphysema, and a small wound on the rostral maxilla communicating with the paranasal sinuses (Figure 1A). Neurological examination was normal, and bilateral nasal airflow was intact. Ophthalmic examination of the right eye revealed absent menace response, normal pupillary and dazzle reflexes, impaired nasolacrimal drainage, moderate exophthalmos, ocular hypertension, ptosis of the upper eyelid related to auriculopalpebral nerve paralysis as well as blepharoedema, mucous discharge, diffuse corneal oedema, and a superficial corneal laceration. The intraocular structures were otherwise normal. Transpalpebral ocular ultrasonography (Toshiba Aplio‐500 ultrasonography unit with 10 MHz linear ultrasound transducer, Toshiba Medical Systems Corporation) revealed hyperechoic material in the vitreous.

**FIGURE 1:**
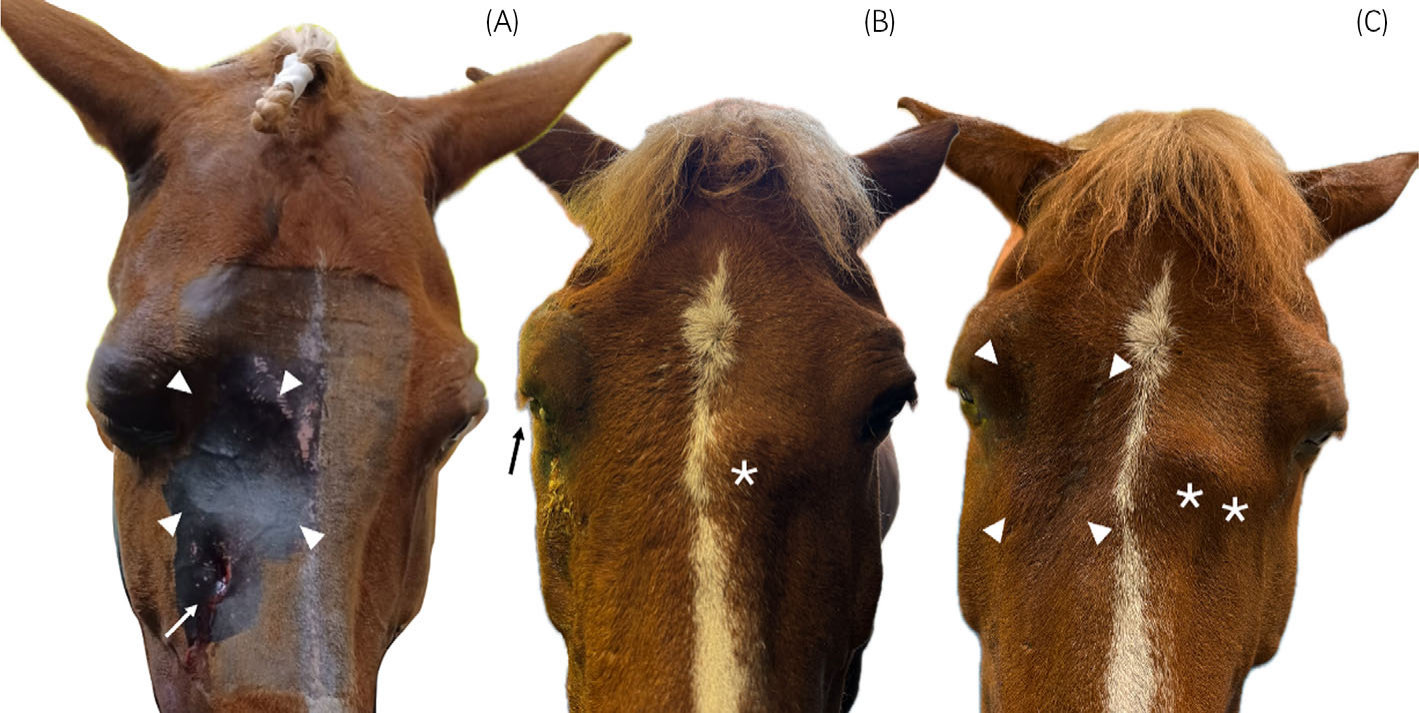
(A) A 14‐year‐old Haflinger mare before the surgical repair of a right‐sided open, depressed (arrowheads) and comminuted facial fracture involving the sinus system and the medial aspect of the orbit. The fracture was open (arrow) with communication to the sinus system. (B) Appearance of the facial contour 10 weeks postoperatively. A nearly symmetrical restoration of the facial contour compared to the contralateral side could be observed. Moderate periocular oedema, ptosis (arrow), purulent dehiscence of the infected wound, as well as suture periostitis in the region of the nasofrontal suture (asterisk) are visible. (C) Skull appearance 1 year post‐surgery. Depression of the skull in the region of the repositioned frontal/nasal bone fragment associated with bone resorption (arrowheads), as well as a progression of the suture periostitis (asterisk), could be observed.

### 2.2 Preoperative CT‐findings

Standing CT‐examination of the head (Canon Medical Systems, 32‐slice detector, 135 kV, 370 mA, 1 mm slice thickness, field of view 50 cm) revealed an open, depressed and severely comminuted skull fracture on the right side involving the frontal, nasal, lacrimal and maxillary bone, sinus system and the medial aspect of the orbital rim and wall (Figure 2A,B). The fracture extended over a 15 cm long and 10 cm wide area with marginal involvement of the caudal left nasal bone and the nasofrontal suture. The dorsomedial orbital rim was displaced ventromedially, and the medial orbital wall laterally, impinging on the right eye. The dorsoventral orbital diameter (defined as the widest dorsoventral distance) was preserved, while the nasotemporal diameter (defined as the widest nasotemporal distance) was reduced by 2 cm compared to the contralateral side (Figures 2A and 3A). Blood accumulation and multiple bone fragments were present in all right‐sided sinus compartments (Figure 4A,D,G).

**FIGURE 2:**
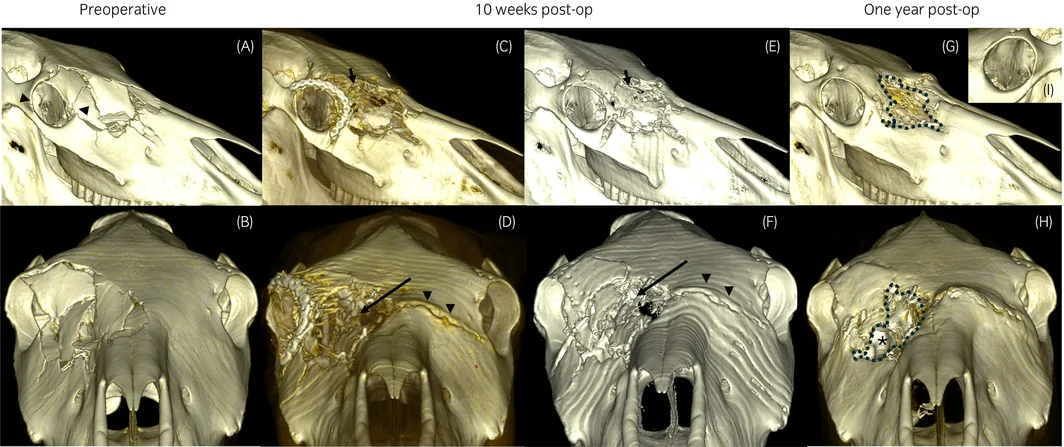
Three‐dimensional computed tomography reconstructions of the skull obtained immediately before and after surgical repair. Preoperatively (A, B), ventral and medial displacement of the dorsomedial orbital rim, together with lateral displacement of the medial orbital wall, resulted in impingement of the right eye. The nasotemporal diameter (arrowheads) of the affected orbit was reduced by approximately 2 cm compared with the contralateral side. Skull appearance immediately before (C, D) and after (E, F) implant removal. Note the ventral displacement of the large frontal/nasal bone fragment caused by pull‐out of the suture wires placed at its caudal margin (arrows). Suture periostitis is evident 10 weeks postoperatively (arrowheads) (C–F). One year post‐surgery (G, H), complete healing of the right orbital rim and wall is visible, with restoration of the nasotemporal diameter comparable to the contralateral side (I). Note the complete resorption of the large frontal/nasal bone fragment and adjacent smaller fragments (dotted area), resulting in recurrent contour depression and exposure of the infraorbital canal (asterisk) in this region.

**FIGURE 3:**
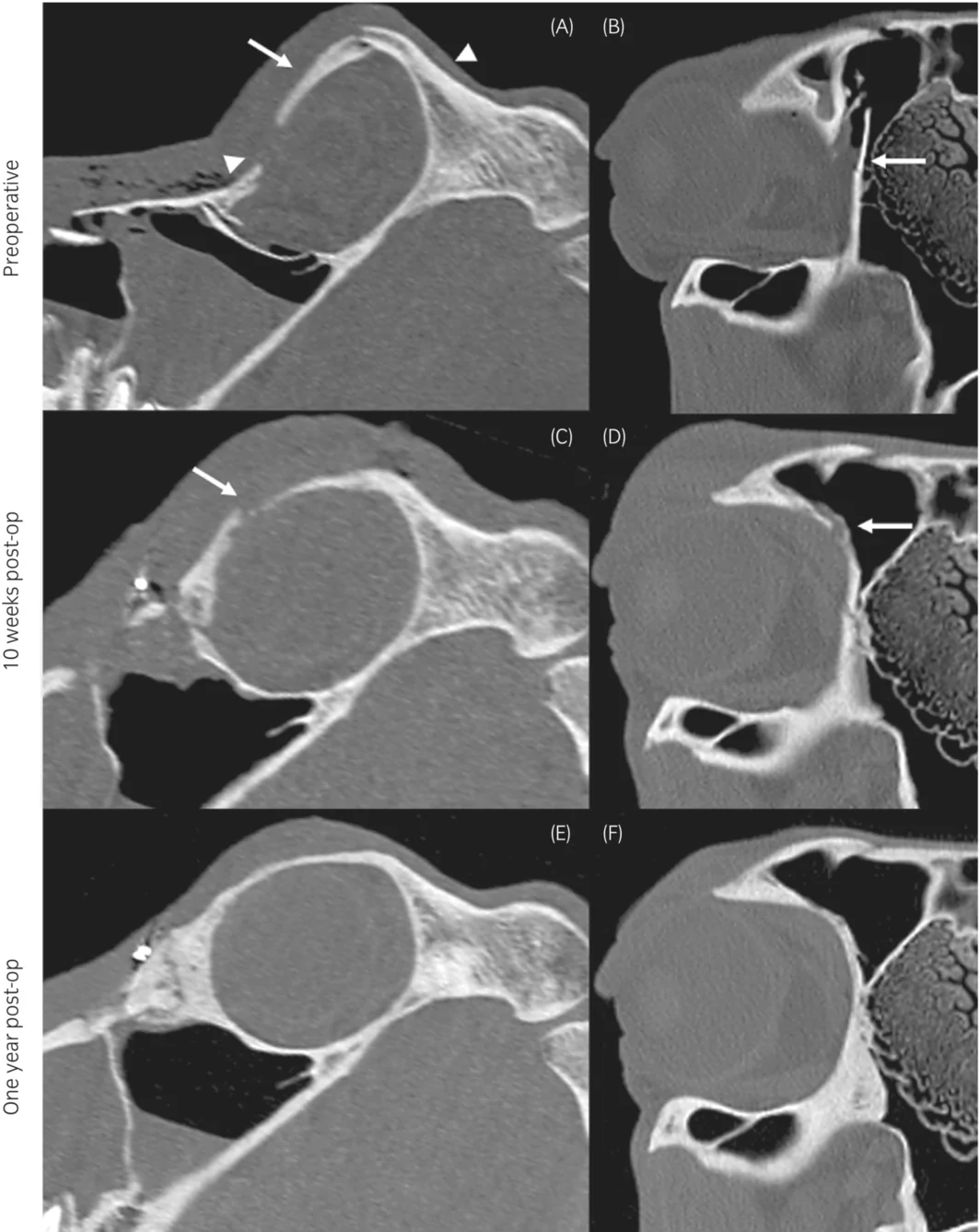
Computed tomography scan centred at the orbital region (A, C, E: Sagittal plane; B, D, E: Frontal plane). The right side (B, D, E) and rostral side (A, C, E) are to the left. Preoperatively (A, B), severe displacement of the orbital rim and the medial orbital wall (arrows) are visible, causing impingement of the right eye. Note also the reduction of the nasotemporal diameter (arrowheads) of the orbit. Ten weeks post‐surgery (C, D), a progressive healing of the orbital rim and wall, and callus formation at the remaining fracture margins (arrows) can be detected. One year post‐surgery (E, F) a complete healing of the right orbital rim and wall with restoration of the preoperative reduced nasotemporal orbital diameter can be observed.

**FIGURE 4:**
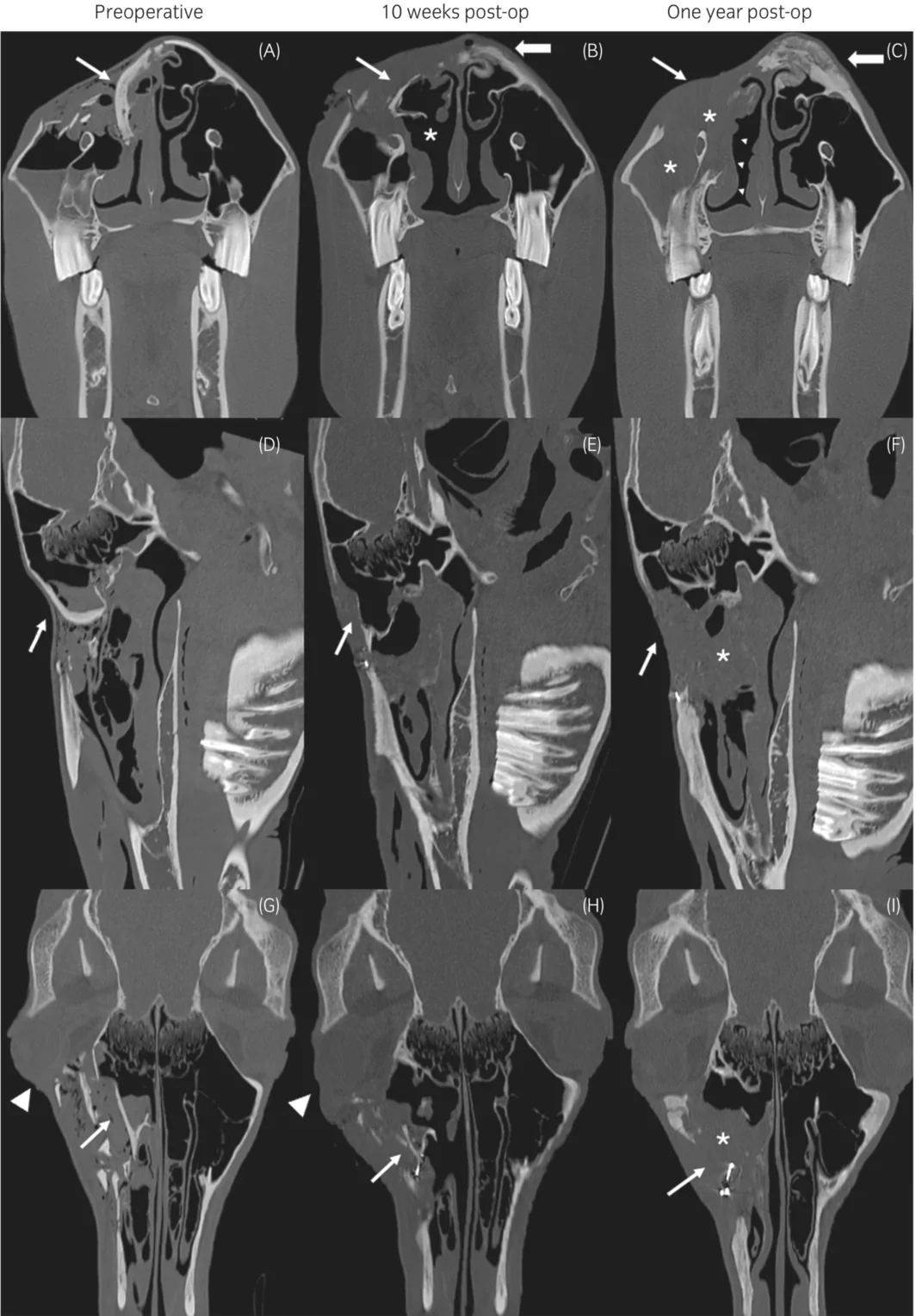
Computed tomography scan centred at the fractured region (A, B, C: Frontal plane level of 110–210 teeth; D, E, F: Sagittal level between the lingual surface of the molars and the nasal septum; G, H, I: Dorsal plane level immediately dorsal to the infraorbital channel). The right side is to the left. On the preoperative scan (A, D, G), a displaced frontal/nasal bone fragment containing the bony part of the nasolacrimal duct (arrows) is evident, as well as exophthalmos (arrowhead). At 10 weeks postoperatively (B, E, H), the large frontal/nasal bone fragment is partially repositioned and shows the first evidence of resorption (arrows), and no exophthalmos (arrowhead) is detectable. A large communication between the nasal passages and the right sinus system (asterisk) as well as new bone formation (large arrow), indicating suture periostitis, could be additionally observed. One year postoperatively (C, F, I), the frontal/nasal bone fragment and the adjacent fragments had completely resorbed, creating a large hole in the right sinus system covered by soft tissue (arrows). A total opacification of the right rostral maxillary sinus is visible (asterisk). The communication between the nasal passages and the right sinus system is completely occluded by a thin ossified membrane (arrowhead), and suture periostitis has progressed (large arrow).

Additional injuries included comminuted fractures of the bony nasolacrimal duct, nasal septum, and axial wall of the dorsal and ventral conchal sinuses at the level of the maxillary septal bulla, creating a large communication between the right dorsal and middle nasal passages and the sinus system (Figures 2A,B and 4A,D,G).

### 2.3 Preoperative treatment

The surgery was postponed for 2 days until soft tissue swelling decreased. This time was used for the development of a detailed surgical plan based on the CT‐examination. During this time, the mare received systemic medical treatment consisting of gentamicin sulphate (6.6 mg/kg IV. once daily, Genta, CP‐Pharma Handelsgesellschaft mbH), Benzylpenicillin‐procaine (Procaine‐Penicillin‐G; 20.000 I.U./kg bwt i.m. once daily, WDT‐Wirtschaftsgenossenschaft deutscher Tierärzte eG) and Flunixin‐meglumine (Flunidol; 1.1 mg/kg bwt i.v. twice daily, CP‐Pharma Handelsgesellschaft mbH). Topical medication of the right eye included atropine (Atropin‐POS‐0.5%, URSAPHARM) and dorzolamide/timolol (Cosopt, dorzolamide 20 mg/mL, timolol 5 mg/mL, Santen) eye drops three times daily, as well as gentamicin (gentamicin‐POS, URSAPHARM) and autologous serum eye drops six times daily, also for 2 days.

### 2.4 Surgery

Flunixin‐meglumine (Flunidol; 1.1 mg/kg bwt i.v.) was administered before sedation. The horse was sedated with xylazine (Xylavet, 0.08 mg/kg bwt i.v., CP‐Pharma Handelsgesellschaft mbH) and butorphanol (Butorgesic; 0.025 mg/kg bwt i.v., CP‐Pharma Handelsgesellschaft mbH), and general anaesthesia was induced with midazolam (Midazolam‐Ratiopharm; 0.25 mg/kg bwt i.v., Ratiopharm GmbH) and ketamine (Ketamin; 2.2 mg/kg bwt i.v., CP‐Pharma Handelsgesellschaft mbH). General anaesthesia was maintained with isoflurane (Isofluran CP; minimum alveolar concentration 1.0, CP‐Pharma Handelsgesellschaft mbH). The horse was subsequently intubated and positioned in left lateral recumbency.

The right ocular region and conjunctival sac were rinsed with polyhexanide/betaine solution (ProntoVet, Braun‐Vet‐Care GmbH), a subpalpebral lavage system (Eye Lavage Kit, Mila International, Inc.) was placed in the lateral aspect of the lower eyelid, and a temporary tarsorrhaphy was performed. The surgical region was aseptically prepared and draped routinely.

A 10 cm long curved skin incision was made in the orbital rim region, starting 2 cm lateral and rostral to the supraorbital foramen and ending 3 cm medial to the facial crest of the zygomatic bone. After transection of the external palpebral musculature, the fracture site was exposed. Depressed fragments were elevated and stabilised using a pre‐shaped 12‐hole 3.5‐mm LCP‐reconstruction‐plate secured with two 12 mm long 3.5‐mm cortical and six 12 mm long 3.5‐mm locking‐head screws (Figure 5). The type and size of the plate, as well as the screw length, were determined based on the preoperative CT‐examination. Preoperative three‐dimensional CT‐reconstructions and a horse skull of comparable size were used to pre‐shape the plate with bending insets. The plate was first anchored to intact regions of the orbital rim, supraorbital (zygomatic process of the frontal bone) and infraorbital (temporal process of the zygomatic bone), to the main medially located fracture region (Figures 2A,B, 5 and 6A). Once stable anchoring was achieved, the depressed fragments of the frontal, lacrimal, and zygomatic bones were elevated with Hohman and Langenbeck retractors and periosteal elevators and simultaneously secured to the plate with cortical and locking‐head screws. Due to the characteristics of the fracture, it was not possible to achieve solid plate‐bone contact along the entire length of the implant. Therefore, locking‐head screws were predominantly used for plate fixation to increase angular stability between the plate, screw and bone. A final repositioning occurred after the cortical and locking‐head screws were tightened, elevating the fragments to the level of the plate (Figure 6B). A separate depressed fragment of the lacrimal bone located rostrally to the plate was elevated as described above and fixed to the previously stabilised frontal and zygomatic orbital rim using cerclage wire (Figure 6B). Therefore, 2‐mm diameter drill holes were made in the opposing edges of the fracture fragments, and one 1‐mm cerclage wire suture was placed between the lacrimal and zygomatic as well as lacrimal and frontal bones.^8^ The surgical site was flushed and closed in two layers: a simple continuous pattern was used for the subcutis (USP 2‐0 polyglecaprone 25), and a simple interrupted pattern was used for the skin (USP 0 polypropylene).

**FIGURE 5:**
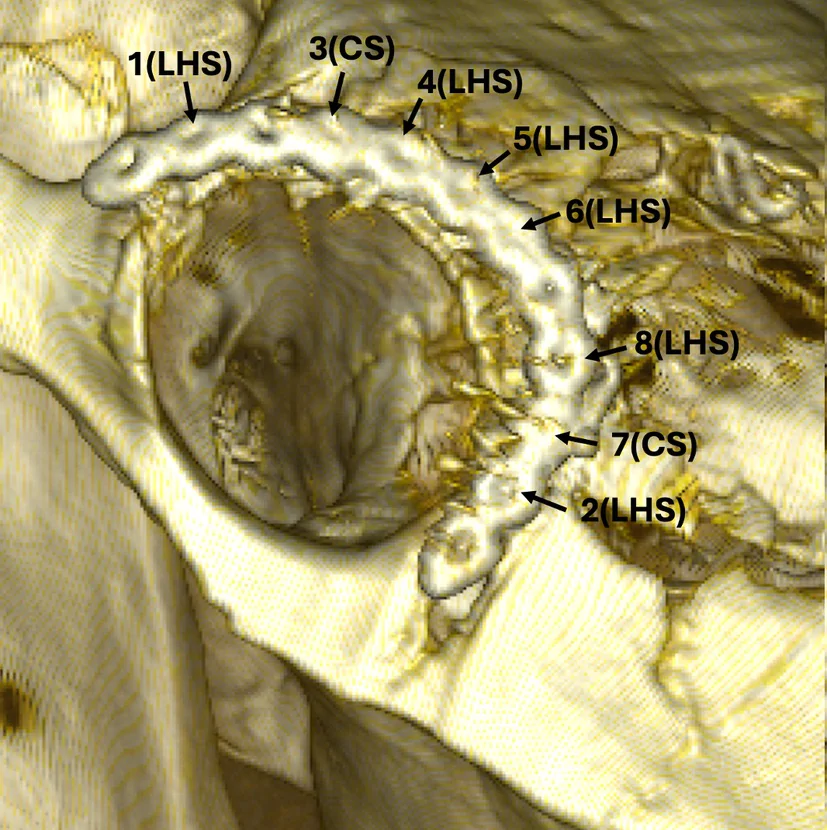
Close‐up of the three‐dimensional computed tomography reconstruction post‐surgical repair shown in Figure 2C, with the detailed location and order of the used 12 mm long 3.5‐mm cortical (CS) and locking‐head screws LHS.

**FIGURE 6:**
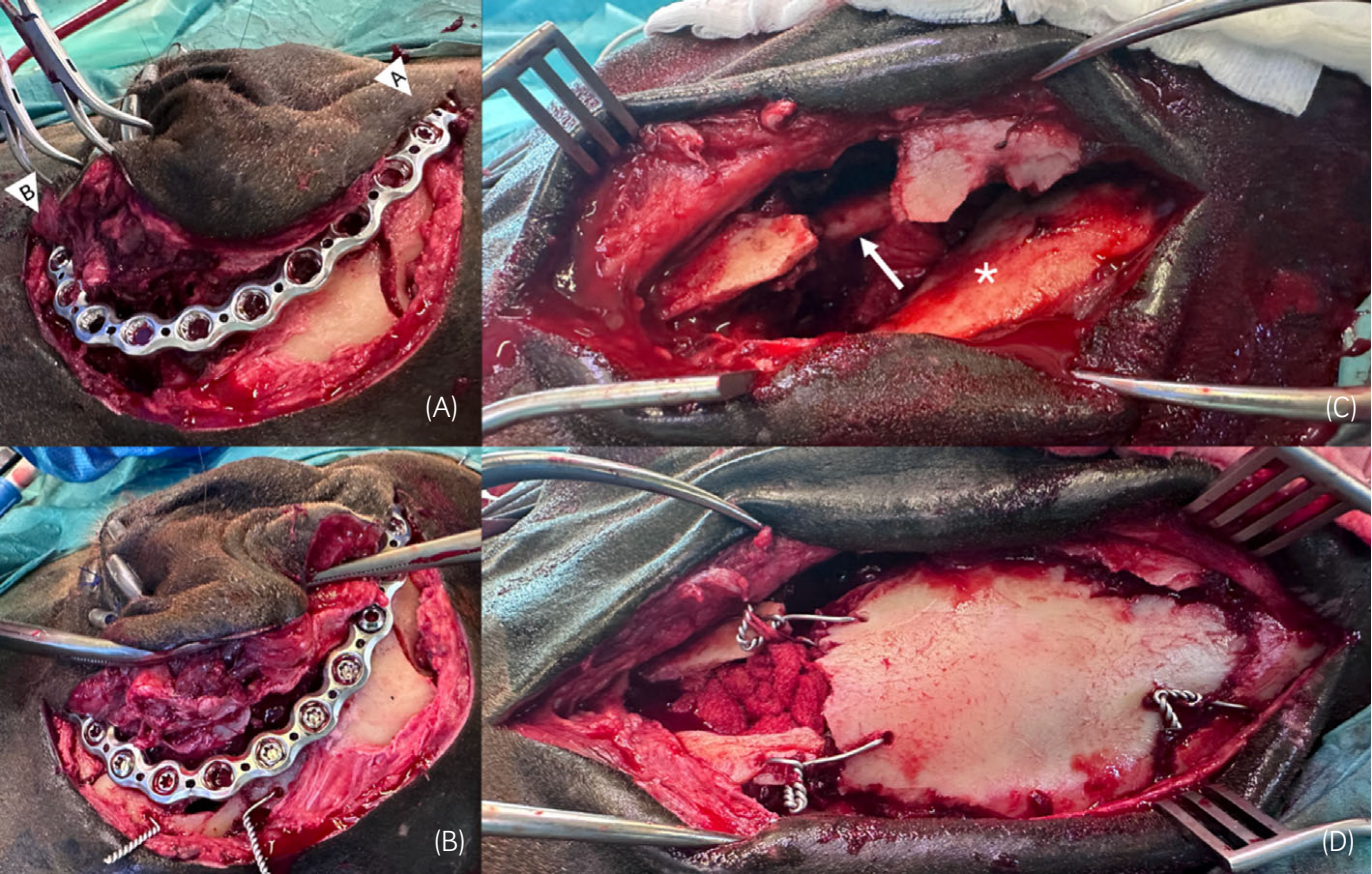
Intraoperative image shows fixation of a 12‐hole 3.5‐mm LCP‐reconstruction‐plate to the non‐fractured parts of the orbital rim, supraorbital (zygomatic process of the frontal bone) (arrowhead A) and infraorbital (temporal process of the zygomatic bone) (arrowhead B) to the main medially located fracture region (A). Appearance after fragments elevation/reposition in the orbital rim region using plate and screw fixation, as well as cerclage wires (B). Intraoperative appearance of the region, with an 8 cm long and 6 cm wide loose frontal/nasal bone fragment (asterisk) severely displaced in the sinus system. Note the complete absence of the periosteum as well as the exposure of the infraorbital channel (arrow) (C). The right sinus system was packed with sterile gauze, and the fragment was fixated with cerclage wires (D).

To expose the axially located fractured fragment, a second 15‐cm full‐thickness incision was made on the right side, extending from the nasofrontal suture to the rostrally located wound; the latter was revised during the procedure. A large, completely loose frontal/nasal bone fragment (Figure 6C) was removed temporarily and wrapped in blood‐impregnated swabs. Sinus lavage, debridement of loose bone, necrotic mucosa, and clots were performed. After the sinus was packed with gauze to control the haemorrhage. Removal of the large bone fragment was avoided, and it was instead stabilised with cerclage wires, as removal would have resulted in a large defect within the sinus system lacking bony coverage. The cerclage wire placement and wound closure were performed in the same manner as for the orbital rim fracture (Figure 6D).

### 2.5 Postoperative care

The sinus tamponade was removed on day 2 post‐surgery and daily lavage with sterile saline was continued until nasal discharge resolved (day 10). Systemic anti‐inflammatory treatment was continued until discharge from the hospital (day 42), while the topical eye medication prescribed preoperatively was maintained until healing of the corneal laceration and normalisation of intraocular pressure was achieved (day 9). An SSI with wound dehiscence and implant exposure was detected in the region of the plate on day 14 post‐surgery and E. coli, resistant to the perioperative antimicrobials (gentamicin sulphate, Benzylpenicillin‐procaine) was cultured from a bacteriological swab collected from the exposed plate. Systemic antibiotics were discontinued, and antiseptic wound lavage was performed with hypochlorous solution (Vetericyn.VF plus, Innovacyn, Inc.) twice daily. The frontal/nasal wound healed uneventfully. At discharge on day 42, moderate ptosis associated with auriculopalpebral nerve paralysis and periocular oedema, as well as low‐grade wound dehiscence with seropurulent discharge and exposure of the plate, were still present.

### 2.6 Follow‐up and plate removal

At 10 weeks postoperatively, the SSI persisted with a visually and palpably stable fracture fixation and with partial improvement of ptosis related to reduction in periocular oedema and improvement of the auriculopalpebral nerve paralysis. A non‐painful swelling was noted along the left nasofrontal suture. The standing CT‐examination performed on the same day confirmed progressive fracture healing, and the infected plate and adjacent cerclage wires were removed directly after under general anaesthesia. The frontal/nasal bone cerclages were left in situ. A second standing CT‐examination 1 day after implant removal, without metallic artefacts, demonstrated progressive healing of the orbital rim and wall, mild periosteal reaction and callus formation at remaining fracture margins (Figure 3C,D). The remaining suture wires at the caudal border of the large frontal/nasal bone fragment appeared to be pulled out, and the fragment was displaced ventrally (Figure 4B,E). Mucosal swelling and sclerosis of bony sinus walls (predominantly the axial surface of the maxillary and zygomatic bones) were present in the right sinus system, along with low‐grade fluid accumulation, and complete sinus opacification (Hounsfield unit (HU) 15–20) in the right rostral and caudal maxillary sinus. Communication between the nasal passages and the right sinus system persisted (Figure 4B). Moderate new bone formation along the left nasofrontal suture indicated mild suture periostitis (Figures 2D and 4B). Endoscopy of the right nasal passage 1 day after the plate removal confirmed patent communication between the nasal passage and the right sinus system. Since the horse showed no clinical signs of sinusitis and pain related to the suture periostitis despite the imaging findings, it was discharged without additional treatment.

### 2.7 1‐year follow‐up

At the 1‐year follow‐up, the horse had fully returned to its intended use as a pleasure riding horse. Ptosis related to a periocular oedema and auriculopalpebral nerve paralysis had completely resolved, with only occasional mild serous nasal and ocular discharge remaining. Apart from a small transparent corneal scar in the region of the previous corneal laceration and complete obstruction of the nasolacrimal duct, no other abnormalities were detected on ophthalmologic examination. The orbital region remained palpably stable, a depression recurred at the frontal/nasal site and the left nasofrontal suture periostitis progressed (Figure 1C). A standing CT‐examination demonstrated complete healing of the right orbital rim and wall with restoration of the preoperative reduced nasotemporal orbital diameter (Figures 2G,I and 3E,F). Additional CT findings were the resorption of a large frontal/nasal fragment, leaving a defect covered by thickened skin and mucosa. The right rostral maxillary and ventral conchal sinus were completely opacified, while the caudal maxillary, frontal and dorsal conchal sinuses showed partial opacification (HU 15–20). The sinunasal communication was completely occluded by a thin ossified membrane confirmed endoscopically. The left nasofrontal suture showed extensive, irregular new bone consistent with progressive periostitis (Figures 2G,H and 4C). As no clinical discomfort was present, no further treatment was undertaken.

## 3 DISCUSSION

This case report describes the clinical and computed‐tomographic findings of a complex, comminuted open craniofacial fracture involving the orbit and sinus system. The fracture was successfully reconstructed using a pre‐shaped 3.5‐mm locking reconstruction plate, resulting in long‐term functional recovery despite several postoperative complications. The primary objective, to restore orbital integrity and ocular function, was achieved, as evidenced by reestablished orbital dimensions and resolution of ocular hypertension, exophthalmos, and nerve dysfunction.

Although orbital fractures in horses are infrequently reported, surgical reconstruction is generally recommended for displaced, unstable fractures, particularly when bony fragments impinge on the globe or adnexal structures, to preserve ocular function.^1^, ^5^, ^7^, ^12^ For severely displaced and unstable fractures, as well as involvement of the orbit, reconstruction plates are considered the method of choice.^13^ Orbital fractures most commonly involve the region of the zygomatic process of the frontal bone and zygomatic process of the temporal bone,^1^, ^7^ likely reflecting their exposed position on the equine skull. In line with this, surgical reconstruction with plate fixation has only been described for fractures of the temporal aspect of the orbital rim.^2^, ^9^ Although surgical management of medial orbital rim fractures using repositioning and wire fixation has been reported, to the authors' knowledge, fixation of these fractures with a pre‐shaped 3.5‐mm LCP reconstruction plate has not yet been described.^1^, ^10^, ^12^

Intraoperative orientation and fragment repositioning can be challenging in cases with severe comminution.^1^, ^8^ Despite severe comminution and reduced nasotemporal diameter of the orbit, a complete restoration of orbital dimensions was achieved, and only minimal callus formation was evident on CT examination 1 year postoperatively. Preoperative CT imaging enabled accurate fracture assessment, development of a detailed surgical plan and pre‐shaping of the implant as previously described.^9^, ^10^ Due to difficulties maintaining the repositioned fragments in place, the plate was first secured to intact orbital rim segments, and then depressed fragments were elevated to meet the plate. This provided stable anatomical alignment and an effective strategy for the complex orbital reconstruction in this case (Figure 6A,B).

Reposition of the medial orbital wall fractures using an implant sizer/tissue expander placed in the conchofrontal sinus or 3D‐printed implants have previously been reported.^4^, ^12^ Although no fracture repositioning was performed in the region of the medial orbital wall in the present case, the fracture healed without complications. It appeared symmetrical on CT examination 1 year postoperatively compared to the contralateral side (Figures 2G,I and 3F). This outcome may be explained by the comparatively minor comminution and absence of marked medial orbital wall displacement, as well as the stable fixation of the orbital rim achieved with the LCP reconstruction plate.^5^, ^9^

The development of an implant‐associated infection delayed fracture healing, extended hospitalisation, and required implant removal under general anaesthesia. Severe comminution, sinus communication and a long plate were all factors predisposing our case to the development of SSI.^10^, ^13^, ^14^ Dowling, Dart and Trope^2^ also described a higher infection rate after facial fracture treatment with plates compared to cerclage wire. As facial bone fractures generally heal faster than limb fractures, implant removal can be safely performed earlier if required, as in the present case. ^2^, ^9^

The use of resorbable implants may represent an alternative in open fractures, as they are associated with lower infection rates and often eliminate the need for implant removal.^15^ However, resorbable materials do not provide the same rigidity as metallic plates,^16^ and it is unlikely that sufficient stability could have been achieved in the present case.

Complete resorption of a large fragment fixated with cerclage wire resulted in a defect of the bony sinus wall covered by well‐healed skin, as confirmed on follow‐up CT‐examination one year postoperatively (Figures 1C, 2H and 4C). In contrast to previous reports,^17^, ^18^ there was no evidence of sequestration or sinocutaneous fistula formation, and the area appeared dense and stable on palpation. Bone resorption is a well‐known complication in human cranioplasty, but has not been described previously in equine skull fracture repair. Disruption of periosteal attachment and, therefore, blood supply, severe comminution and instability caused by limited fixation, likely contributed to this outcome.^19^, ^20^ While some reports recommend removal of bone fragments not covered by the periosteum,^1^, ^2^ others describe successful repositioning of a large maxillary bone fragment after complete detachment from the mucosa and periosteum, without subsequent complications.^8^ As the bone fragment was too large to be removed in this case, it was repositioned. Although a subsequent resorption occurred, it initially provided biological support. Several surgical reconstruction procedures for cases with resorption of large bone fragments are described in human medicine.^21^ As these methods are not currently applicable in equine surgery, and the cosmetic outcome was satisfactory for the owners, no further reconstruction procedures were performed in the present case. Techniques, such as rotational periosteal flap to improve the fragment's blood supply,^22^ or intrasinus expandable devices for enhanced stability,^10^, ^12^ might help to prevent this complication.

The non‐painful progressive suture periostitis most likely developed as a consequence of the initial trauma—given the close proximity of the suture line to the fracture—and may have been exacerbated by ongoing micromotion associated with an incompletely stable fixation.^23^, ^24^ Some authors recommend internal fixation in such cases.^13^ However, due to the absence of pain and complicated procedures that had already been performed, further surgery was ruled out.

A predisposition for secondary sinusitis has been reported in association with comminuted orbital fractures.^7^ Despite the severe fracture, postoperative sinusitis was limited, possibly due to a direct communication between the nasal passages and the right sinus (Figure 4B). One year postoperatively, CT revealed complete opacification of the right rostral maxillary sinus and partial opacification of the frontal and dorsal conchal sinuses, suggestive of mucosal proliferation or a mucocele, a condition reported in human medicine as a long‐term consequence of trauma.^25^, ^26^, ^27^ A definitive distinction of this condition from mucosal thickening is possible by means of contrast‐enhanced CT or MRI, which is not currently used routinely in equine head imaging.^27^, ^28^ The clinical relevance of this finding in the present case, in the absence of any signs of discomfort, remains uncertain. In human reports, mucoceles have been associated with potential complications such as extension into adjacent structures or secondary infection.^26^

## 4 CONCLUSION

Despite several case reports, the literature regarding repair and long‐term healing characteristics of complicated facial and orbital bone fractures in equines remains limited.^1^, ^10^, ^12^ In the present case, excellent reconstruction of the orbit using an LCP‐reconstruction‐plate was achieved despite severe comminution and surgical site infection. Although CT is now widely used for preoperative evaluation of equine head diseases, there are no reports about postoperative evaluation of fracture repair using this modality, in contrast to the established standards in human medicine.^6^, ^26^ Based on CT follow‐up, two important postoperative findings are reported for the first time in this case. This includes complete resorption of a large bone fragment without evidence of sequestration or sinocutaneous fistula formation and extensive sinus opacification without clinically apparent sinusitis. This modality should be considered standard not only for preoperative assessment of facial fractures but also for long‐term postoperative monitoring of healing in horses. Bone resorption and mucosal formation should be recognised as further possible complications experienced in horses with facial bone fractures. While these findings were not associated with clinical impairment in this report, their clinical relevance warrants further investigation in a larger case series.

## FUNDING INFORMATION

There was no funding.

## CONFLICT OF INTEREST STATEMENT

The authors have declared no conflicting interests.

## AUTHOR CONTRIBUTIONS

**A. Kalinovskiy:** Writing – original draft; writing – review and editing; conceptualization; investigation. **C. Lischer:** Supervision; writing – review and editing; investigation; conceptualization. **J. C. Eule:** Investigation; supervision. **K. Thieme:** Investigation. **K. Mählmann:** Writing – original draft; writing – review and editing; supervision.

## DATA INTEGRITY STATEMENT

Andrey Kalinovskiy had full access to all the data in the study and takes responsibility for the integrity of the data and the accuracy of data analysis.

## ETHICAL ANIMAL RESEARCH

Research ethics committee oversight not required by this journal: retrospective study of clinical records.

## INFORMED CONSENT

The horse's owners provided written consent.

## Data Availability

The data that support the findings of this study are available upon reasonable request from the corresponding author. Open data sharing exemption granted by the editor.
